# Preliminary evidence about irritability in patients with epilepsy treated by perampanel as first add‐on therapy compared to levetiracetam and valproic acid

**DOI:** 10.1111/cns.13098

**Published:** 2019-01-23

**Authors:** Claudio Liguori, Katherine Turner, Francesca Izzi, Martina Assogna, Maria P. Canevini, Nicola B. Mercuri, Fabio Placidi

**Affiliations:** ^1^ Epilepsy Centre Department of Systems Medicine University of Rome “Tor Vergata” Rome Italy; ^2^ Epilepsy Centre San Paolo Hospital Milan Italy; ^3^ IRCCS Fondazione Santa Lucia Rome Italy

**Keywords:** epilepsy, irritability, levetiracetam, perampanel, valproic acid, I-EPI, effectiveness, 12-month follow-up

## Abstract

**Aims:**

Irritability has been described as a frequent adverse event in patients affected by epilepsy and treated with perampanel (PER), levetiracetam (LEV), and less frequently with valproic acid (VPA). Since the questionnaire for irritability (I‐EPI) is a validated instrument to measure this psychiatric manifestation in patients affected by epilepsy, in this study we aimed at investigating the effect of PER as first add‐on therapy on I‐EPI. Moreover, we compared the effectiveness and I‐EPI scores obtained at 12‐month follow‐up visits in patients treated by PER, LEV, or VPA in order to measure irritability as a consequence of these treatments.

**Methods:**

We collected data from 17 patients treated by PER, 16 patients treated by LEV, and 16 patients under VPA treatment followed for 12 months.

**Results:**

We did not document significant changes of I‐EPI questionnaire between baseline and follow‐up in the PER group. As concerning the comparison of I‐EPI among PER, LEV, and VPA groups, we documented lower global scores in PER than both LEV (*P* < 0.05) and VPA (*P* < 0.05) groups. Moreover, patients under PER treatment showed lower scores than LEV and VPA (*P* < 0.05) in I‐EPI items measuring the gentle personality, anxiety of having epileptic seizures in front of others, and irritability in thinking that they can have an epileptic seizure.

**Conclusions:**

This retrospective study described a stable and possibly lower degree of irritability in patients starting PER than LEV and VPA treatments, although we documented the comparable effectiveness of PER, LEV, and VPA as first add‐on treatments in patients affected by uncontrolled epileptic seizures. However, the small sample of patients included in this study and the absence of I‐EPI scores obtained at baseline visits in LEV and VPA groups require further investigations to confirm this preliminary evidence.

## 1 INTRODUCTION

Patients affected by epilepsy not only have to manage epileptic attacks but also comorbid medical and/or psychiatric problems.^1^ In the past years, literature focused on depression and anxiety as important factors for determining quality of life and daily stress in patients affected by epilepsy.^2^ However, behavioral problems, such as irritability, also affect patients with epilepsy. Irritability is a mental state of extreme sensitivity to any kind of stimulation, which can produce different physical and mental responses.^3^ In epilepsy, irritability is intended as a multidimensional construct, which can be measured by a specific and validated questionnaire (I‐EPI). It consists of 18 items measuring the possibility to feel irritability in different situations.^4^ Several external factors may rouse irritability in patients affected by epilepsy, such as the stigma and social discriminations due to epileptic condition, the irregular and unpredictable nature of their seizure, and the side effects of antiepileptic drugs (AEDs). Accordingly, several AEDs have shown irritability as a psychiatric side effect in patients affected by epilepsy.^5^, ^6^, ^7^ Valproic acid (VPA), levetiracetam (LEV), and perampanel (PER) are three drugs owing respectively, to the first, second, and third generation of AED. Although all the three drugs have been associated with the occurrence of irritability as side effect, VPA has been recently associated with significant less irritability than other AEDs, such as LEV.^5^

Briefly, PER was recently licensed for the treatment of focal and generalized epilepsies.^8^, ^9^ It is a noncompetitive α‐amino‐3‐hydroxy‐5‐methyl‐4‐isoxazole proprionic acid (AMPA) receptor antagonist demonstrated efficacious in focal and generalized seizures in randomized controlled trials and clinical real‐life studies.^10^, ^11^, ^12^ LEV is a widely used AED, featured by a high therapeutic index and wide margin of safety compared to other AEDs.^13^ VPA is one of the oldest AED, characterized by a significant efficacy in treating epileptic seizures, but associated with several side effects.^14^ Taking into account the literature describing the real‐life use of the aforementioned AEDs, on the one hand it has been described that PER is associated with the occurrence of irritability, which has been mainly observed at high doses and in patients with intellectual disability.^15^, ^16^, ^17^ On the other hand, LEV and VPA have been both described causing irritability or psychiatric manifestations as side effects, frequently related to epileptic seizure control.^5^, ^6^, ^18^ As a special condition characterized by the resolution of ictal and inter‐ictal epileptic discharges associated with the occurrence of psychiatric manifestations, the term “forced normalization” has been postulated, and this rare manifestation has been documented in patients treated by LEV or VPA.^18^, ^19^

Hence, since less but increasing data about the real‐life use of PER treatment, also as first add‐on, have been published in the recent past,^20^, ^21^ this retrospective observational study aimed at better describing the effectiveness of PER and its psychiatric side effects, focusing on irritability. On these bases, the primary objective of the present study was to measure irritability by using the I‐EPI questionnaire in patients affected by uncontrolled seizures who start PER as first add‐on treatment at baseline and 12‐month follow‐up visits. The second objective of this study was to compare I‐EPI questionnaire obtained at follow‐up in patients treated by PER, VPA, or LEV as first add‐on treatment in order to monitor irritability in all the three groups. Finally, we compared the effectiveness of these three AEDs used as first add‐on treatments at the 12‐month follow‐up visit.

## 2 METHODS

The present report is a retrospective observational study based on individual charts review of patients affected by uncontrolled seizures and under approved monotherapies, who started LEV, VPA, or PER as first add‐on AEDs for better controlling their secondarily generalized seizures. Data were collected by the two centers participating to the study, the Epilepsy Centre of the University Hospital of Rome “Tor Vergata,” and the Epilepsy Centre of the San Paolo Hospital in Milan. The eligible patients were retrospectively selected among those receiving LEV, VPA, or PER as first add‐on therapy from September 2016 to June 2017 and followed for 12 months. Patients were classified according to the 1981 International League Against Epilepsy, which was in use when patients were diagnosed.^22^ We collected and analyzed data considering the baseline visit in which the AED was proposed as first add‐on therapy and the following visit at 12‐month follow‐up.^23^ We included patients who maintained PER, VPA, and LEV treatment for 12 months, excluding those who interrupted the treatment for major adverse events. Since the primary objective of the study was to measure irritability by using the I‐EPI questionnaire in patients affected by epilepsy starting PER as first add‐on therapy, exclusively in this group of patients the I‐EPI questionnaire score was achieved at baseline and follow‐up visits. Unfortunately, the I‐EPI questionnaire scores were obtained only at follow‐up visits in the VPA and LEV groups and used for the comparison among the three groups (PER *vs* LEV *vs* VPA). Considering the retrospective nature of the study, the following data were collected: age, sex, time since epilepsy onset, etiology (symptomatic or cryptogenic epilepsy), history of psychiatric disorders, 1‐month total seizure count at baseline and 12 months after starting LEV, VPA, or PER, previous AED monotherapy, I‐EPI questionnaires scores at the 12‐month follow‐up visit. Titration was performed according to clinical practice for PER, VPA, and LEV. For the statistical analysis, we analyzed and compared these data among the three groups: (a) 50% responder rate, defined as the percentage of patients obtaining a minimum of ≥50% seizures reduction in seizures’ frequency compared to baseline, (b) seizure freedom (considered as absence of seizures between time points), (c) I‐EPI questionnaire scores.

The I‐Epi is a 18‐item self‐administered questionnaire. Patients have to rate their irritability level on a 6‐point likert scale format, ranging from “never” (1), “almost never” (2), “rarely” (3), “sometimes” (4), “often” (5) to “always” (6). The overall irritability score was obtained by summing single domain scores and ranges from 18 to 108; higher numerical values reflect a higher severity of symptoms. The answers to question 3 were reversed in calculation of the overall scores.

The 18 items of the I‐EPI questionnaire identified four main domains related to irritability in patients affected by epilepsy: Physical Functioning, Verbal Functioning, Temperamental Functioning, and Epilepsy Functioning. Scores in each item and the global scores were compared among the three groups.

The statistical analysis was performed using commercial software Statistica 10.0 program, Statsoft Inc, Tulsa, OK, USA.^24^ Descriptive data were expressed as mean and standard deviation for quantitative analyses. The one‐way analysis of variance (ANOVA) was used to compare descriptive data among the three groups and p value was set at *P* < 0.05 for statistical significance. The paired *t* test was used to compare I‐EPI data between baseline and follow‐up in PER group, and between patients in the PER group who were seizure‐free and not seizure‐free at follow‐up. The Bonferroni correction was applied when appropriate. This study was approved by the local Ethics Committee of the University Hospital of Rome “Tor Vergata”.

## 3 RESULTS

Forty‐nine patients affected by uncontrolled secondarily generalized seizures who started a first add‐on treatment with LEV, VPA, or PER were included in this retrospective analysis. No patient was affected by learning or intellectual disabilities. PER was administered as first add‐on AED in 17 patients, whereas 16 patients started LEV and 16 patients started VPA as first add‐on AED (see Table 1). The three groups did not significantly differ in terms of demographic data; moreover, groups did not differ for seizures’ baseline frequency, disease duration, age of epilepsy onset, history of psychiatric disorders, and previous AEDs (see Table 1). Eleven patients treated with PER, eight patients treated with LEV, and 12 patients treated by VPA showed an unremarkable brain MRI and epilepsy was defined as cryptogenic; on the other hand, six PER patients, eight LEV patients, and four VPA patients were affected by symptomatic epilepsy since they showed brain MRI alterations. Finally, two PER, two LEV, and three VPA patients presented history of psychiatric diseases.

**Table 1:** Demographic and clinical data of patients

	LEV (n = 16) (mean±SD)	VPA (n = 16) (mean±SD)	PER (n = 17) (mean±SD)	*P* Value
Age (*years*)	37.2 ± 10.2	31.9 ± 8.6	37.7 ± 18.5	NS
Sex	8F8M	10F6M	8F9M	NS
Etiology	8 cryptogenic 8 symptomatic	12 cryptogenic 4 symptomatic	11 cryptogenic 6 symptomatic	NS
Disease Duration (*months*)	19.6 ± 15.0	17.3 ± 12.7	15.1 ± 9.7	NS
History of Psychiatric Disorders	2/16	3/16	2/17	NS
Previous AED monotherapy	11 CBZ 3 LTG 1 OXC 1 TPM	6 CBZ 4 TPM 2 OXC 2 ZNS 2 LTG	7 CBZ 4 ZNS 3 OXC 2 LTG 1 PB	NA
Seizures at baseline (*per month*)	2.1 ± 0.9	2.4 ± 3.5	3.2 ± 2.1	NS
Seizures at follow‐up (*per month*)	0.7 ± 0.9	1.0 ± 1.9	1.0 ± 1.6	NS
AED Dose mg/daily	2000.0 ± 1028.7	806.7 ± 469.4	5.6 ± 2.1	NS

LEV, levetiracetam; VPA, valproic acid; PER; perampanel; f, female; m, male; SD, standard deviation; CBZ, carbamazepine; LTG, lamotrigine; OXC, oxcarbazepine; TPM, topiramate; ZNS, zonisamide; PB, phenobarbital; NA, not admitted; NS, not significant; AED, antiepileptic drug.

**Figure 1:**
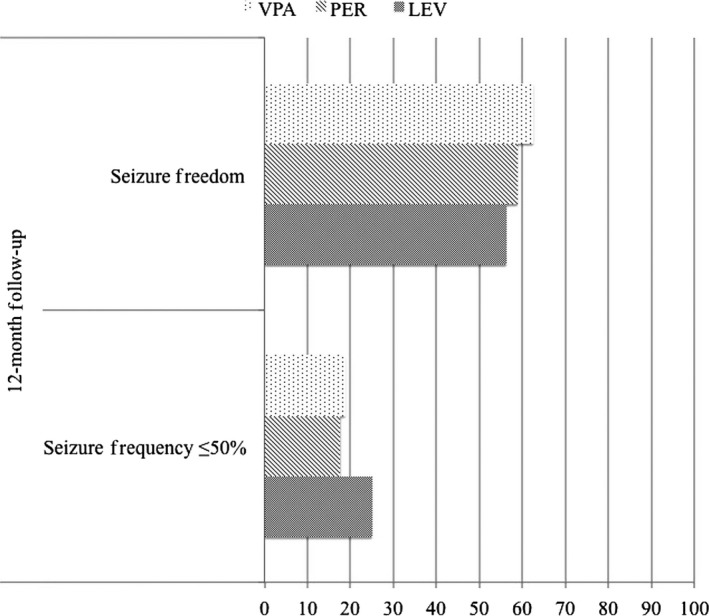
Effectiveness data in LEV, PER, and VPA groups. *(PER: perampanel; VPA: valproic acid; LEV: levetiracetam)*

Analyzing data achieved at 12 months follow‐up among the three groups, we documented the similar efficacy of PER, LEV, and VPA considering seizure freedom or seizures reduction ≥50% (see Figure 1).

Considering I‐EPI questionnaires, comparing data obtained at baseline and follow‐up in PER patients we did not document significant differences in the global score (48.43 ± 21.43 vs 44.47 ± 16.56, *P* > 0.05) and in the single items’ scores. Notably, comparing the I‐EPI total scores completed at follow‐up visits by all patients, we documented lower scores in PER group compared to both VPA and LEV groups (see Table 2). Moreover, the items 3, 15, and 18 (owing to “I have a gentle personality,” “I get anxious if I have an epileptic seizure in front of others,” and “I get very irritable thinking that i can have an epileptic seizure,” respectively) were lower at follow‐up in the PER group compared to both LEV and VPA groups (see Table 2). Finally, patients treated by PER showed lower scores than LEV in I‐EPI subitem 6 and 8, quantifying the possibility to raise the voice if somebody contradicts them (“if somebody contradicts me, I raise my voice”) and considering the belief that others attain success more quickly than them (“I believe that others attain success more quickly than I do”) (see Table 2). We also compared in the group of patients treated by PER the I‐EPI questionnaires obtained at the follow‐up visit between seizure‐free and not seizure‐free patients, and we exclusively documented the significance for the item 3 score of the I‐EPI questionnaire (see Table 3).

**Table 2:** I‐EPI questionnaire scores at follow‐up visits

	LEV (n = 16) (mean ± SD)	VPA (n = 16) (mean ± SD)	PER (n = 17) (mean ± SD)	*P* Value
Item 1	2.7 ± 1.5	2.5 ± 1.5	2.2 ± 1.4	NS
Item 2	3.5 ± 1.5	3.0 ± 1.6	2.6 ± 1.3	NS
Item 3	4.5 ± 1.5	4.1 ± 1.6	2.6 ± 1.5	PER vs LEV < 0.01 PER vs VPA < 0.01
Item 4	2.7 ± 1.2	3.1 ± 1.6	2.3 ± 1.4	NS
Item 5	2.0 ± 1.5	2.1 ± 1.5	1.7 ± 1.0	NS
Item 6	3.8 ± 1.0	3.7 ± 2.0	2.6 ± 1.2	PER vs LEV < 0.01
Item 7	3.4 ± 1.7	3.0 ± 1.6	2.9 ± 2.2	NS
Item 8	3.2 ± 1.8	3.0 ± 1.9	2.0 ± 1.5	PER vs LEV < 0.01
Item 9	3.6 ± 2.2	3.9 ± 1.6	3.8 ± 1.8	NS
Item 10	1.2 ± 0.6	1.9 ± 1.9	1.9 ± 1.6	NS
Item 11	2.7 ± 2.1	2.7 ± 1.8	1.8 ± 1.2	NS
Item 12	3.1 ± 2.2	3.7 ± 1.4	2.9 ± 2.0	NS
Item 13	3.2 ± 1.2	3.7 ± 1.6	2.6 ± 1.5	NS
Item 14	2.9 ± 1.7	3.4 ± 1.6	2.8 ± 1.5	NS
Item 15	4.3 ± 1.8	4.4 ± 1.5	2.3 ± 1.8	PER vs LEV < 0.01 PER vs VPA <0.01
Item 16	2.4 ± 1.8	3.0 ± 1.7	3.0 ± 1.9	NS
Item 17	2.7 ± 1.7	2.9 ± 1.7	1.9 ± 1.3	NS
Item 18	4.0 ± 1.9	4.4 ± 1.7	2.5 ± 2.1	PER vs LEV < 0.01 PER vs VPA < 0.01
Global Score	56.1 ± 16.5	58.4 ± 14.8	44.5 ± 16.6	PER vs LEV < 0.01 PER vs VPA <0.01

LEV, levetiracetam; VPA, valproic acid; PER; perampanel; I‐EPI, questionnaire for irritability in epilepsy; SD, standard deviation; NS, not significant.

**Table 3:** I‐EPI questionnaire scores in PER subgroups

	Seizure‐free (n = 10) (mean±SD)	Not seizure‐free (n = 7) (mean±SD)	*P* Value
Item 1	2.1 ± 1.5	1.9 ± 1.4	NS
Item 2	2.8 ± 1.3	2.2 ± 1.2	NS
Item 3	3 ± 1.5	2.0 ± 1.2	0.03
Item 4	2.9 ± 1.5	1.9 ± 1.1	NS
Item 5	2.3 ± 1.3	1.6 ± 1.4	NS
Item 6	3.2 ± 1.5	2.7 ± 1.6	NS
Item 7	3.5 ± 2.3	3.1 ± 2.7	NS
Item 8	2.8 ± 2.4	2.4 ± 2.4	NS
Item 9	4.4 ± 2.1	4.2 ± 2.8	NS
Item 10	2.8 ± 3.0	2.7 ± 3.1	NS
Item 11	2.9 ± 3.0	2.6 ± 3.5	NS
Item 12	4.0 ± 3.3	3.6 ± 3.8	NS
Item 13	3.5 ± 3.5	3.9 ± 3.8	NS
Item 14	4.0 ± 3.7	3.9 ± 4.3	NS
Item 15	3.2 ± 4.1	4.2 ± 4.8	NS
Item 16	4.2 ± 4.4	4.6 ± 4.9	NS
Item 17	3.3 ± 4.8	3.7 ± 5.5	NS
Item 18	3.4 ± 5.1	5.0 ± 5.6	NS
Global Score	44.5 ± 17.0	40.1 ± 16.1	NS

PER; perampanel; I‐EPI, questionnaire for irritability in epilepsy; SD, standard deviation; NS, not significant.

## 4 DISCUSSION

The present retrospective observational study investigated the symptom of irritability in patients starting PER as first add‐on treatment for better controlling their drug‐resistant seizures, also compared to patients starting VPA and LEV as first add‐on AED. Moreover, we assessed the effectiveness of PER, LEV, and VPA after 12‐month follow‐up. The main result of this study was the unmodified I‐EPI questionnaire between baseline and follow‐up in patients affected by epilepsy who started PER as first add‐on AED. Next, at follow‐up I‐EPI scores were lower in the PER group compared to both LEV and VPA groups. Considering the single items of the I‐EPI questionnaire, scores of items 3, 15, and 18 were lower in the PER group than VPA and LEV groups, thus documenting less irritability in the personality trait and in social conditions of daily living in patients who were prescribed PER than VPA and LEV. Moreover, patients in the PER group also showed lower scores at I‐EPI items 6 and 8 than LEV. Nevertheless, PER was not associated with significant changes of I‐EPI questionnaire between baseline and follow‐up visits, it showed a comparable effectiveness to VPA and LEV at the 12‐month follow‐up visit. In the study design, we hypothesize to use LEV and VPA as comparators of PER, since LEV may induce psychiatric adverse events, whereas VPA is less frequently associated with irritability as a psychiatric adverse event than other AEDs.^5^ Hence, I‐EPI scores were collected in patients who were visited at 12‐month after the prescription of PER, LEV, or VPA.

These findings may broaden our view about the need of measuring irritability by using validated instruments, as I‐EPI questionnaire. In keeping with the results of this study, it is conceivable that PER may less frequently cause irritability at lower doses when used as first add‐on treatment. This result concords with a previous report published by our group and describing a good tolerability profile of PER when used as first adjunctive treatment.^20^ Furthermore, recent real‐life observations confirmed the tolerability and effectiveness of PER also in highly drug‐resistant patients with different epilepsy syndromes.^21^ In agreement with this recent evidence, serious psychiatric adverse events were usually reported by patients with psychiatric comorbidities; moreover, it appeared much more evident that PER may be more efficacious in treating symptomatic epilepsy, as previously suggested considering the mechanism of action of the drug.^21^, ^25^

Irritability is a psychiatric symptom occurring in almost 60% of patients affected by epilepsy.^17^ Several circumstances including socioeconomic, demographic, and clinical factors may be correlated with irritability. Since it has negative effects on daily living, an instrument able to quantify and monitor this symptom has been validated and provided. The I‐EPI questionnaire represents a specific measure of irritability in clinical and research practice.^4^ We documented that PER did not change the I‐EPI questionnaire in our patients’ population. This finding may be also explained considering the evidence that irritability is usually more frequent in patients under three or more AEDs.^4^, ^17^ Moreover, level of irritability is closely related to seizure control and with AED doses; in particular, a post hoc analysis of behavioral side effects related to PER clearly described that irritability could be dose‐dependent and more frequent in patients with previous history of psychiatric disorders.^17^, ^26^ Thereafter, the good effectiveness of PER and its use as first add‐on therapy in our experience can explain the stability of I‐EPI questionnaire scores and the lower level of irritability in PER patients compared to VPA and LEV patients. Notably, irritability is usually associated with psychiatric diseases 27 and the scarce presence of psychiatric comorbidities in our patients’ population may explain the low level of irritability found in the PER group. We also compared the subgroups of patients ‐month follow‐up visit to patients treated by PER who continued to experientreated by PER and seizure‐free at the 12ce seizures at follow‐up, but we did not document significant differences between the two groups, although the trend in higher I‐EPI scores in patients who were seizure‐free at follow‐up. However, the small sample of patients did not permit to drive definitive evidence and further studies should be performed. Hence, the possible explanation of our findings could consist in the fact that PER was prescribed as first add‐on AED and the therapeutic dose was lower than the dose often reached in more refractory epileptic patients.^8^, ^28^, ^29^

Our study has several limitations: the retrospective design, which can imply a bias in patients’ selection; the relatively short observation period of 12 months; the small number of patients included, although the number of patients in the three groups was similar thus allowing a comparison with statistical significance. Finally, we can monitor the change in I‐EPI questionnaire exclusively in patients starting PER, since I‐EPI questionnaire was not administered at baseline in patients under VPA and LEV. However, this first clinical retrospective investigation comparing irritability at 12‐month follow‐up visits in patients under PER, VPA, or LEV as first add‐on AED proposes the possibility to monitor irritability during clinical visits in the epilepsy centers in order to better measure this disabling symptom, which can also represent a psychiatric adverse event in patients affected by epilepsy. Hence, our conclusions should be cautiously considered as a preliminary impression, which requires further validation from studies on larger populations.

## 5 CONCLUSIONS

Although irritability is a frequent symptom in patients affected by epilepsy, it can be controlled in patients starting low doses of PER as first add‐on treatment for uncontrolled epileptic seizures. The possible positive influence of PER on irritability can also be explained by the good effectiveness demonstrated by the drug, which was comparable to that of VPA and LEV when used as first add‐on therapy. This preliminary evidence needs to be further confirmed by prospective and controlled longitudinal studies.

## CONFLICTS OF INTEREST

Claudio Liguori has been a consultant and/or attended to scientific advisory board for Eisai. Dr. Fabio Placidi received Research Support from EISAI Pharmaceuticals. Dr. Francesca Izzi has been a consultant and/or attended to scientific advisory board for Eisai. Nicola Biagio Mercuri, Martina Assogna, Katherine Turner and Maria Paola Canevini declare no conflict of interest or financial disclosures.

We confirm that we have read the Journal's position on issues involved in ethical publication and affirm that this report is consistent with those guidelines.
